# Utilization of Rituximab for Refractory Rowell Syndrome

**DOI:** 10.1155/2021/2727382

**Published:** 2021-07-29

**Authors:** Sukhraj Singh, Sandra Sheffield, Nisha Chowdhury, Swetha Nuthulaganti, Zareen Vaghaiwalla, Karishma Ramsubeik

**Affiliations:** ^1^Division of Rheumatology, Department of Medicine, University of Florida Health Jacksonville, Jacksonville, FL, USA; ^2^Division of Internal Medicine, Department of Medicine, University of Florida Health Jacksonville, Jacksonville, FL, USA

## Abstract

Rowell syndrome describes the occurrence of erythema multiforme-like lesions in patients with cutaneous lesions of lupus erythematosus. The clinical picture of atypical erythema multiforme-like lesions, presence of chilblains, speckled ANA pattern, anti-Ro/SSA, or anti-La/SSB antibodies, and absence of infectious or pharmacologic triggers in a patient with systemic lupus erythematosus are some of the classic clinical and serologic features. Histopathologic and serologic findings can help differentiate this process from erythema multiforme. We present a case of young woman with systemic lupus erythematosus, end-stage renal disease due to lupus nephritis, and a remote history of Steven–Johnson syndrome due to sulfa allergy who presented to the hospital with a recurrent, progressive, targetoid erythematous rash involving more than 60% of her body surface area. Our patient had several hospitalizations in the recent past for this erythematous rash and had failed oral therapy with prednisone 1 mg/kg and hydroxychloroquine. In view of the minimal improvement and increasing severity and patient exhibiting early features of mast cell activation syndrome, the patient was treated with pulse intravenous glucocorticoids followed by rituximab with an excellent response. We highlight a unique case report of progressive Rowell syndrome refractory to standard of care with an excellent response to rituximab.

## 1. Background

Rowell syndrome (RS) is a rare clinical presentation first described in 1963 by Dr. Neville Rowell and colleagues as an association between systemic lupus erythematosus (SLE) and erythema multiforme (EM) [1]. The defining immunologic features include a speckled antinuclear antibody (ANA) pattern, anti-Ro/SSA or anti-La/SSB antibody, and positive rheumatoid factor (RF) [1]. Since its inception, less than 100 cases have been reported [2, 3]. Among these cases, a higher incidence of cases have been reported in women, with a ratio of women to men 8 : 1. Some cases have been linked to drug exposures, while others manifested with an initial presentation of SLE. Reported cases of RS often fluctuate in clinical and histological presentations and do not always meet the criteria, contributing to the debate on whether RS is its own distinct entity or simply the coexistence of two separate disease processes. Management of RS often overlaps with cutaneous lupus erythematosus and includes topical and oral steroids, antimalarials, immunosuppressive drugs, and pain management.

## 2. Case Presentation

A 26-year-old African American female with a medical history of SLE, ESRD due to LN, remote history of Steven–Johnson Syndrome due to sulfa allergy, and resistant hypertension presented to the emergency department with painful, peeling, blistering, rash and fever. She reported that the rash developed over a period of 3 months with an intermittent response to prednisone therapy. Three weeks prior to presentation, the rash became progressively worse. She developed fevers came on one week prior to presentation. The rash started on her arms and progressively spread. She admitted associated shortness of breath accompanied by a productive cough with green, bloody sputum, as well as nausea and vomiting. Patient reported compliance with her home dose of prednisone but may have not taken her mycophenolate mofetil regularly. She missed two sequential hemodialysis sessions due to pain with walking from the blistering rash on her soles of the feet.

**Figure 1:**
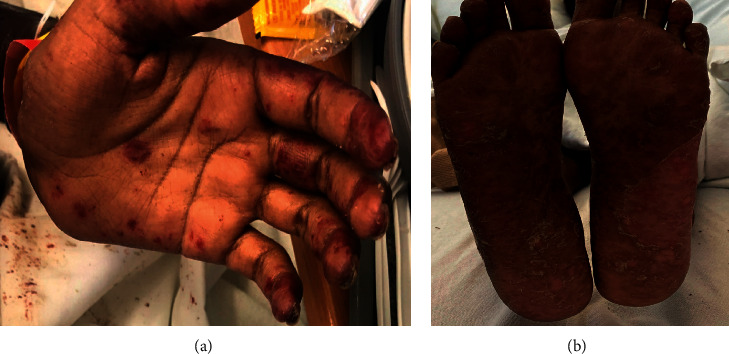
Chilblain lupus erythematosus (LE), deep red to violaceous plaques on palms and soles. Some appear annular targetoid pain and pruritis.

**Figure 2:**
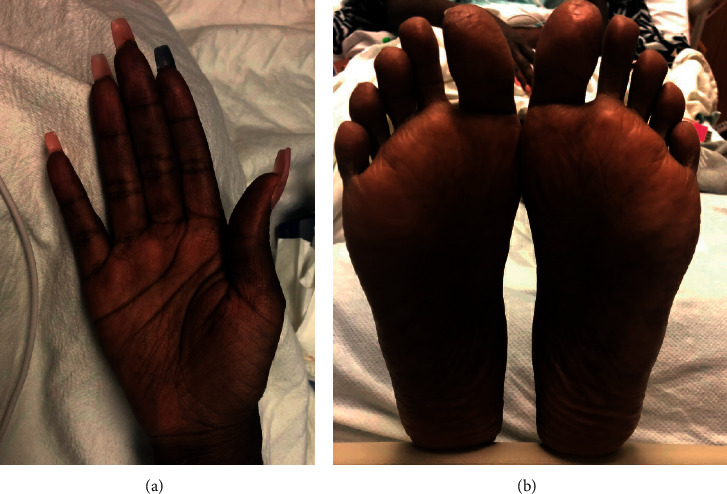
Complete resolution of lesions seen in Figure 1 approximately 3 months after treatment.

**Figure 3:**
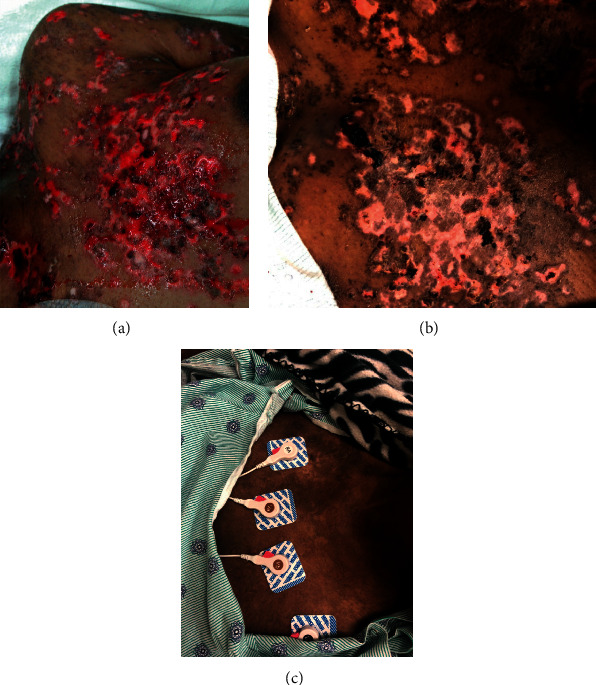
Left anterior chest wall demonstrating full-thickness epidermal necrosis with large areas of bleeding, denuded skin. Center image shows healing two weeks posttreatment and the image on the left shows complete resolution at 3 months since induction with rituximab.

Her physical exam was significant for fever of 100.6°F and tachycardia with heart rate of 130 beats per minute. Skin exam was evident for brown plaques on the forehead and bilateral cheeks with sparing of the nasolabial folds, with erosions and hemorrhagic crust involving the philtrum, ears, forehead, nose, chin, and lips. There were dark brown plaques with few erosions and atrophic scars on bilateral forearms and lower legs with deep red to violaceous painful targetoid lesions on the palms and plantar surfaces (Figures 1 and 2). The skin over the trunk had pink eroded macules, and there were pink patches on the back within a background of dusky necrotic plaques. There were multiple full-thickness epidermal necroses with large areas of bleeding with the denuded skin on the arms and chest in the sun exposed distribution (Figure 3). Oral mucosae were negative for any oral lesions.

Patient was found to be pancytopenic with white blood cell count (WBC) 2.03 (Ref: 3.4–10.8 × 10^3^/UL), absolute neutrophil count (ANC) of 1.25 (Ref: 1.4–7.0 × 10^3^/UL), hemoglobin (Hb) 8.8 g/dL (Ref: 11.1–15.9 g/dL), hematocrit (Hct) 28.9% (Ref: 34–46%), platelet 69 (150–450 × 10^3^/UL), sodium 138 mmol/L, potassium 3.9 mmol/L, chloride 98 mmol/L, CO_2_ 21 mmol/L, BUN 41 mg/dL (Ref: 6–20 mg/dL), Cr 5.49 mg/dL (Ref: 0.57–1.0 mg/dL), calcium 7.2 mg/dL (8.7–10.2 mg/dL), albumin 3.2 g/dL, phosphorous 5.0 mg/dL (Ref: 1.2–2.2 mg/dL), AST 286 IU/L (Ref 0–40 IU/L), ALT 31 IU/L (0–32 IU/L), total bilirubin 0.5 mg/dL, alkaline phosphatase 136 IU/L, CK 91 U/L, serum lactate was 0.80 mmol/L, and ferritin was 5885 ng/mL (Ref: 15–50 ng/mL). Electrocardiogram was consistent with sinus tachycardia. Chest X-ray did not show any lung infiltrates or effusions. Ultrasound of the abdomen did not show an acute abdominal process, and the liver demonstrated normal echogenicity, echotexture, and size; the spleen was not enlarged. Sputum gram stain and cultures were unremarkable. Blood cultures were negative for bacteria and yeast. Patient was anuric due to chronic dialysis. Evaluation of lupus activity showed double-stranded DNA (dsDNA) antibody 42 IU/mL (Ref: 0–9 IU/L). Complement 3 (C3) was 43 mg/dL (Ref: 90–180 mg/dL) and complement 4 (C4) was 9 mg/dL (Ref: 10–40 mg/dL). Rheumatoid factor was negative. Sjogren's anti-SSA was >8.0 AI (Ref: 0–0.9 AI) and Sjogren's anti-SSB was <0.2 AI (Ref: 0–0.9 AI). HIV screen was negative, and viral hepatitis panels for hepatitis A, B, and C were negative. Skin biopsies showed focal interface changes, and periodic acid Schiff (PAS) stain was negative for fungal microorganisms. Gram stain highlighted surface cocci bacteria. VZV and herpes I/II immunostains were negative. The direct immunofluorescence revealed that finely granular deposition of C3, IgM, and fibrinogen is consistent with lupus.

Given her presentation of a fever, shortness of breath, and productive cough, there was a concern for postinfectious erythema multiforme. The nucleic acid amplification tests for multiple respiratory pathogens such as *Mycoplasma*, influenza A, influenza H1, influenza H3, influenza A virus H1 2009, influenza B, respiratory syncytial virus, parainfluenza virus type 1, 2, 3, 4, human metapneumovirus, rhinovirus/enterovirus, adenovirus, *Chlamydia pneumoniae*, and *Mycoplasma pneumoniae* were negative. Blood cultures did not grow any bacteria or fungal organisms. Skin wound cultures were also negative for any organisms except for skin flora. Historically, there were no preceding infections reported. Our presumptive primary diagnosis was acute on chronic cutaneous lupus with epidermal necrolysis due to noncompliance with medications; differentials included Rowell syndrome, bullous lupus, Steven–Johnsons syndrome, and toxic epidermal necrolysis (TEN). Hemophagocytic lymphohistiocytosis/mast cell activation syndrome (HLH/MAS) was also a concern given pancytopenia, fevers, elevated transaminases, and elevated ferritin. However, after further evaluation by hematology, HLH/MAS was less likely given halving of ferritin after first dose of intravenous steroids, lack of hepatosplenomegaly, lack of elevation in bilirubin and triglycerides, patient did not have persistent fevers, and soluble IL-2 receptor was 2021 pg/mL (Ref: 175.3–858.2 pg/mL), needed to be greater than 2400 pg/mL (greater than 2 standard deviations of reference range) to meet criteria, and clinical picture was also not as critical as usually seen. The clinical picture of atypical EM-like lesions, presence of chilblains, speckled ANA pattern, elevated anti-Ro/SSA, and absence of infectious or pharmacologic triggers in a patient with SLE confirmed our suspicion for Rowell syndrome.

Due to the extensive skin surface area involvement, the patient was treated with vancomycin intravenously for primary and secondary skin infection. Gram-negative coverage was held due to severe allergy to penicillin and cephalosporin antibiotics. The pancytopenia was suspected due to an acute flare of systemic lupus erythematosus, and the patient was initially started on methylprednisolone 1.5 mg/kg intravenously daily. Given more than 60% of body surface area (BSA) involvement, the patient was transferred to the burn unit for extensive debridement and multifaceted wound care. Mycophenolate mofetil was held given the elevated transaminases. Pulse doses of intravenous methylprednisolone were escalated to 1000 mg intravenous daily for three days given the concern for acute cutaneous lupus erythematosus with epidermal necrosis and concomitant MAS. After normalization of transaminases, negative testing for latent tuberculosis and hepatitis B and C, and negative blood cultures, the patient was given rituximab 375 mg/m^2^ intravenously at day 0 and day 15 with excellent maintenance of remission of skin disease and cytopenias. This dose was chosen by the treating provider, given patient being dialysis dependent, thus more immunosuppressed state at baseline and recent elevation in transaminases.

It was reassuring that early suspicion for MAS appeared steroid-responsive to pulse intravenous methylprednisolone. The soluble IL-2 receptor alpha measurement was not elevated, and serum ferritin halved after the first dose of pulse steroids. Despite the improvements in serum and systemic symptoms, the skin manifestations were slow to respond. During 2-week and 12-week follow-up of the patient, there has been maintenance of clear skin without any active cutaneous lupus manifestations. Systemic Lupus Erythematosus Disease Activity Index 2000 (SLEDAI-2K) was 0 at 12-week follow-up.

## 3. Discussion and Conclusion

Erythema multiforme is an acute, immune-mediated condition characterized by the appearance of distinctive target-like lesions on the skin often accompanied by erosions or bullae involving the oral, genital, and/or ocular mucosae [4]. EM has very well-known causes in the literature including infections (herpes simplex virus or *Mycoplasma pneumoniae* infection), medications (nonsteroidal anti-inflammatory drugs (NSAIDs), antiepileptics, and antibiotics), malignancy, and autoimmune diseases [4]. Classical EM is precipitated by trigger factors such as infective agents or drugs and is not associated with any specific serological antibodies or with chilblains. The rare overlap of EM and SLE and the presence of serological criteria can help make the diagnosis of Rowell syndrome [1].

Rowell syndrome is a rare entity that has been described with all subtypes of systemic lupus erythematosus. Beck and Anderson first described unique and distinct features associated with Rowell syndrome [5]. These characteristics include coexistence of SLE, erythema multiforme-like lesions, and immunological changes. The first described unique characteristics include positive tests for rheumatoid factor, speckled pattern antinuclear antibody, and precipitating antibodies to saline extract of human tissue (anti‐SjT) now known as La/SSB and Ro/SSA antibodies [6]. The erythema multiforme-like lesions are painful, pruritic, annular, erythematous, and red to violet plaques with blisters. The lesions are mainly seen in the arms and legs and less frequently on the face, neck, chest, and mouth. They could last from a few days to over a month [1]. Histopathologic findings of RS are controversial as well. The presence of necrotic keratinocytes is not specific to RS because it might be found in 54% of subacute cutaneous lupus erythematosus (SCLE) lesions [7]. The direct immunofluorescence of RS and EM exhibits similar findings to include deposits of C3 and immunoglobulin M in the dermal capillaries [6, 7]. Like SLE, Rowell syndrome is believed to be an autoimmune disease with an interplay of genetics, hormones, and environment factors. There are no specific autoantibodies associated with Rowell syndrome.

Since its first description in 1963, there has been controversy whether RS is a separate entity or a coincidental occurrence associated with cutaneous lupus erythematosus [1]. Approximately 95 cases have been described in the literature since it was first described [3]. We summarize the multiple different diagnostic criteria proposed over the last five decades (Table 1). Diagnostic criteria proposed by Torchia et al. seem to be the most comprehensive [8].

The response to treatment is variable, and frequent recurrences have been reported [7]. Different therapies studied for RS have produced variable results [7, 9, 10]. Various therapeutics options have been described for the management of RS including hydroxychloroquine, chloroquine, azathioprine, dapsone, thalidomide, and corticosteroid therapy [10]. Most RS patients respond well to the first-line therapies outlined above. Our patient continued to have breakthroughs of EM-like rash and progression despite the use of hydroxychloroquine and steroids. The development of early mast cell activation syndrome (MAS) in our patient with worsening cutaneous disease also warranted implementation of more powerful immunosuppression. We report the first case of persistent Rowell syndrome and LE nonspecific skin disease, which is treated with rituximab to maintain remission.

Rituximab is an anti-CD20 monoclonal antibody approved for the treatment of non-Hodgkin lymphoma, chronic lymphocytic leukemia, and rheumatoid arthritis, but it is used off-label for many other conditions [11]. Studies have shown its effectiveness in treating autoimmune skin disorders, such as pemphigus vulgaris, pemphigus foliaceus, vasculitis, SLE, and SCLE [12]. Currently, there are seventeen cases of cutaneous manifestation of SLE and eight cases with refractory SCLE treated with rituximab [12, 13]. Of the eight refractory SCLE cases, seven showed effective results except one. The dosing regimen was varied from 1 g every 2 weeks to 375 mg/m^2^ weekly for 4 weeks [12]. Maintenance doses varied from every 2 months to 1 year. Although rituximab has shown effective results in treatment of SCLE, it has not been effective for chronic cutaneous lupus erythematosus. Rituximab therapy has been reported in treatment of five cases of erythema multiforme major [14]. However, these patients differed from the one presented in this case, as they did not have SLE and suffered from severe, chronic erythema multiforme major with positive antidesmoplakin antibodies in four of the five cases [14]. In other studies, 2 patients have been reported with LE nonspecific condition of vasculitis which had complete resolution of mucocutaneous disease after rituximab [15].

Rowell syndrome has a good prognosis with most cases reporting complete remission in one year; however, there are some cases citing recurrence of disease and up to two years until remission [16]. In the case presented, pulse intravenous glucocorticoids were used to induce remission, and two doses of rituximab 375 mg/m^2^ two weeks apart were implemented to maintain remission. Although rituximab has been utilized as off-label treatment of SLE, this may be the first documented case of treatment of Rowell syndrome with rituximab.

## Figures and Tables

**Table 1:** Summary of proposed diagnostic criteria for Rowell syndrome by different authors.

	Diagnosis
Rowell et al. [5]	All features required
LE	
EM-like lesions (with the absence of any known precipitating factors)	
Speckled pattern of ANA	
Anti-SJT antibody (anti-La/SSB)	
Positive rheumatoid factor	

Lee et al. [10]	All features required
LE	
EM-like lesions (with the absence of any known precipitating factors)	
Chilblains	
Speckled pattern ANA	
Anti-La/SSB antibody	
Positive rheumatoid factor	

Zeitouni et al. [1]	3 major + 1 minor criteria
Major criteria	
SLE, DLE, or SCLE	
EM-like lesions (with/without involvement of mucous membranes)	
Speckled pattern ANA	
Minor criteria	
Chilblains	
Anti-Ro/SSA or anti-La/SSB	
Positive rheumatoid factor	

Torchia et al. [8]	Presence of all 4 major + 1 minor criteria
Major criteria	
Presence of CCLE (DLE and/or chilblain)	
Presence of EM-like lesions (typical or atypical targets)	
At least one positivity among speckled ANA, anti-Ro/SSA, and anti-La/SSB antibodies	
Negative DIF on lesional EM-like lesions	
Minor criteria	
Absence of infectious or pharmacologic triggers	
Absence of typical EM location (acral and mucosal)	
Presence of at least one additional ARA criterion for diagnosis of SLE besides discoid rash and ANA and excluding photosensitivity, malar rash, and oral ulcers	

## Data Availability

This is a case report of a single patient; to protect privacy and respect confidentiality, none of the raw data have been made available in any public repository. The original reports, laboratory studies, imaging studies, and outpatient clinic records are retained as per normal procedure within the medical records of our institution.

## Abbreviations

SLE: Systemic lupus erythematosus
ESRD: End-stage renal disease
SJS: Steven–Johnson syndrome
TEN: Toxic epidermal necrolysis
EM: Erythema multiforme
LE: Lupus erythematosus
ANA: Antinuclear antibody
SSA: Sjogren's syndrome-related antigen A
SSB: Sjogren's syndrome-related antigen B
RS: Rowell syndrome
MAS: Mast cell activation syndrome
LN: Lupus nephritis
VZV: Varicella zoster virus.
